# Foreign

**Published:** 1840-07-04

**Authors:** 


### FOREIGN.

*On the Treatment of Convulsions occurring during Labour, with an illustrative Case.* (Read before the Medical Society of Manchester, by Joseph A. Smith, Esq., M. R. C. S.)—The process of parturition is usually marked by few deviations from its well-ascertained and ordinary phenomena; and although popular prejudice and sympathy have generally associated notions of peril with the interesting hour of travail, yet the slightest reflection will convince even those who are comparatively ignorant of the details of labour, that the hazard of life has not been wantonly appended to the discharge of that important process. The few cases of difficulty, or of imminent danger, occur as deviations from a general law, and are to be viewed as anomalous exceptions to the rule, which, if not permitting the absence of some suffering, at least is consistent with a general assurance of safety.

Very contradictory statements have been made by systematic writers as to the number of cases of *preternatural* labour which occur in a given ratio. Some of these registers have been transcripts of hospital practice; others, compiled from the private memoranda of individual practitioners. Thus, Dr. Hagen, of Berlin, states, that out of three hundred and fifty instances he found it necessary to have recourse to the forceps ninety-three times, to the crotchet twenty-eight. Twenty-six of this series died. Dr. Dewees, of Philadelphia, reports, that in more than three thousand cases he has *not met with one* requiring the use of the crotchet. Similar, though not equal, discrepancies might be cited. Our own experience, our familiarity with the practice of friends, and of others, strongly induce the conclusion, that improper and unnecessary interference may fairly be noticed as the baneful yet prolific source of these *unusually* disastrous records.

Deviation from the ordinary process of parturition may be studied under a twofold aspect. There are forms of danger and difficulty, of which only the well-instructed attendant can take cognizance—presentation of the funis, for instance, or malposition of the fœtal head. There are others which are external in their manifestation, instantly evident, and appealing to the observation of the most ignorant bystander. Among these, hæmorrhage and convulsion claim our especial attention.

My present object is the discussion of the question *as to the justifiable application of the long forceps, or the adoption of other modes of artificial delivery, in cases of convulsion occurring during labour at the full period of gestation.* What is the precise condition imperatively demanding interference; and within what latitude of description (adopting such predicament as the extreme instance) ought the surgeon to confine himself in the formation of a class of cases to which instrumental or other aid is applicable?

*Convulsion* is an abstract term, the aggregate expression of results, not of the pathological condition constituting the cause of deviation from ordinary action. Deficiency of circulating blood may produce such condition equally with an overgorged state of the cerebral vessels; hence the absurdity of prescribing for mere symptoms, without reference to the *varying* and even opposite causes on which they not unfrequently depend.

In an essay on Puerperal Convulsions, published in 1818 by Dr. Dewees, the division adopted is—the *epileptic*, the *apoplectic*, the *hysterical* forms of the disorder. In apparent confirmation of the correctness of this arrangement, Dr. Merriman has observed that "the cases that alone are deserving of the appellation of *puerperal convulsion*, which have fallen under his observation, have borne a *very exact resemblance to the epilepsy*."

Resemblance is not necessarily identity. Satisfied that there is no essential difference between the two, so far as is appreciable by differences in the more or less important and prominent symptoms,—if the treatment of epileptic paroxysms were well and thoroughly understood, there would remain no occasion for further or present inquiry. The application of a general principle would insure the correct treatment, not only of the ordinary forms of convulsion, but of that mere modification of the disorder, as witnessed during labour. But have we, upon the slender datum of resemblance, warrant for the institution of such analogy? Imperfectly as the pathology of all disorders of the nervous system is at present understood, is it not highly unphilosophical (to say the least of it) to confound or blend in its definition one disease, of which we know essentially little, with another, of which our knowledge is still more limited? If the division adopted above be deserving our serious consideration, if any definite significancy attach to words, we are compelled to conclude, that if puerperal convulsion be not absolutely identical with epilepsy, the two pass into each other by gradations that are insensible; or at least, that if the assumption be not strictly true, it forms an extremely close approximation to exact definition. Is it true that puerperal convulsion is a mere modification of epilepsy,—that it is nothing more than a paroxysm of this latter obscure affection occurring accidentally, or as excited by some internal or peculiar irritant during labour, in the same way as it has been speciously and plausibly contended that there is nothing essentially peculiar in puerperal fever,—that this is nothing more than ordinary fever of the typhoid type occurring incidentally, and perhaps somewhat modified by the parturient condition? Is it also certain that hysteria occurs during labour, in the same relation? Has dissection confirmed the views of those who have contended that the puerperal convulsion is apoplexy? If these questions were answered in the affirmative, the inquiry would still return—What, then, is epilepsy; what is the precise character of that deviation; *what the pathological predicament on which the symptoms depend?*

It may be urged that these researches would be found in practice more curious than useful, that the symptoms denote something, which if not denominated epilepsy, bears at least (so far as external indications can point its true character) a close resemblance to it; and further, that the remedies which experience has demonstrated to be safe and useful in the one instance, have been freely recommended in the other. Even this is not wholly true; but if, for the sake of argument, it be admitted, at best such reasoning is idle, unscientific, founded on analogies that will feebly bear a close investigation. The question as to the propriety of manual or instrumental interference with the loaded uterus during convulsion, can only be decided by having reference to a closer scrutiny than seeming resemblance between two forms of disorder can supply, of which little or nothing more is known than participation in a symptom common to many forms of irritation of the nervous system.

Irritability of the uterus from distension has been ranked by the highest authority as one of the exciting causes of convulsion. If this be true, then bloodletting may prove a rather questionable remedy; the effects of sudden and very copious depletion closely following an unconscious and large expenditure of sensorial power, may be incorrectly chargeable upon careful removal of the contents of the uterus. I am inclined to think that in those cases admitting of relief from the lancet, the removal of a *moderate* quantity of blood is all that affords the hope of success, further depletion being decidedly injurious, and falsifying our diagnosis as to the seat and nature of the irritation. Recurring to the valuable published lectures of that able and distinguished physiologist and accoucheur, Dr. Blundell, I find him remarking—"Should you ask me in what manner these convulsions are produced, I should reply, that the more probable and immediate cause of them is a pressure on the brain, and perhaps on the spinal marrow also. This pressure results sometimes from an effusion of blood, still more frequently from effused water, and most frequently of all from mere congestion. If you were to ask me, How is it that these congestions of blood are produced? I should reply, not, in most instances, by general plethora, but rather from an increased action of the cerebral vascular system, the carotid arteries, and perhaps the vertebral; indeed, in the adult, if we examine the carotid, we may sometimes see the pulse beating and jumping in the neck. Whether," he observes, "men are but children of a larger growth, I do not stay to inquire; but I am sure that women are, at least in constitution. Now, that the convulsive and hydrocephalic affections of young children are connected with an increased action of the cerebral vessels, is certain." Truly, idiopathic inflammation of the brain or of its investing membranes, is of frequent occurrence, still I think it can be rendered evident that the source of convulsion during labour is as commonly to be found in the uterus, as are some forms of hydrocephalus traceable to irritation of the intestinal mucous lining. The analogy is surely as fair as that which presumes the identity of puerperal convulsion, epilepsy, apoplexy, and hysteria.

The existence of a class of convulsive cases, in which the symptoms are clearly referrible to uterine irritation, does not supersede the possibility of such irritation being sometimes seated primarily in the nervous centre itself. Careful observation will enable us to discriminate accurately between the two cases. The effects of remedies, more especially of the abstraction of blood, ought assiduously to be watched, and if the ordinary methods of subduing cerebral excitement be adopted unsuccessfully, a fair presumption is thereby afforded that the source of the mischief is not located within the head; that some distant, unremoved irritant is defying the lancet, or rendered more destructive, as the energies of the system sink under efficient depletion.

Professor Burns thinks that "puerperal convulsions are quite different from epilepsy, inasmuch as they recur at no future time, except perhaps in a subsequent pregnancy. The principal difference, and one of a highly important nature is, that whilst the *symptoms* are the same in both diseases, they arise in epilepsy from some organic affection of the brain or *direct irritation of that organ*, whilst, in the case in question, they rather depend upon some *sympathetic* and *temporary* cause." Nothing can afford so just an illustration of the conjectural character of the art of medicine as the extreme diversity of opinion prevalent amongst our highest authorities, not merely in reference to purely speculative questions, but in relation even to practical matters of the highest import. I apprehend there are few practitioners unbiassed by prejudice, and unfettered by preconceived opinions or a slavish subservience to some favourite authority, who will refuse their assent to the distinction now sought to be enforced, or deny that it ought most materially to influence our practice. The parturient condition of the uterus is carefully to be recognised. The accompanying irritability of the nervous system aggravates and disturbs still more completely the excited and distended organ; in this way convulsion becomes coexistent with uterine action, and though in many instances of a secondary character, has almost invariably arrested more attention than the forgotten yet primary source of the mischief, the condition of the uterus itself.

Under the influence of convictions such as these, I am glad to observe in Dr. Burns' work the after-admission, that "the patient is suffering from a disease connected with the state of the uterus, and that state is got rid of by terminating the labour." Few practitioners would resort to instrumental or manual interference in any instance without first having recourse on general grounds to the abstraction of blood; whether, with a view of relieving cerebral congestion, or for the sake of rendering the uterus more easily accessible, either motive, or both, would secure and justify its adoption. Careful interference is not to be confounded with rashness, or premature and unskilful efforts. If, with Dr. Hall, we have the requisite condition present or attainable, namely, an easily dilatable and yielding os uteri, if, after the lapse of as much time as the urgency of repeated and violent convulsion will permit, sensibility and consciousness are evidently waning, if the inefficiency of tolerably copious bloodletting have demonstrated the source of danger to be beyond its agency, if in addition to these precautions the intestinal canal has been thoroughly cleared, whether by injection or otherwise, and the head subjected to the evaporation of a cooling wash; then, indeed, the remark of Professor Burns becomes truly orthodox: "Delivery does not indeed always save the patient, or even prevent the recurrence of the fits, *but it does not thence follow that it ought not to be adopted.* I look upon it," says the learned professor, "as indispensable, if the convulsions are not checked by bleeding. In *almost all cases the forceps is requisite*, and turning is rarely required."

I attended, some time ago, a female in labour, whose case made a powerful impression upon my mind at the time; and I relate it, as involving principles of treatment in conformity with the principles just advanced.

*Case.*—M. W., a plump, muscular young woman, about twenty-four years of age, sent hastily for me, in her second confinement. Before proceeding up stairs, I was informed that several hours had elapsed since the first accession of pain, and that the occurrence of convulsion, at first only trifling and transient, but afterwards of a more violent and alarming character, had excited the worst fears for her safety. I learned that the habits of the patient were irregular, and that she had suffered much from mental excitement, consequent upon domestic inquietude. Armed with this information as to the probable predisponent and exciting causes of convulsion, I found her scarcely sensible, struggling with violent grasp, and purple-bloated features; her pulse, as the fits subsided, full, but not materially hardened; the teeth closed; the eyes rolling wildly, except for a few moments, when a disposition to sleep was suddenly arrested by a renewal of convulsive effort. In fact, the natural contractile efforts of the uterus seemed to pass into spasmodic contortion of the whole set of voluntary muscles; the tongue became bitten and protruded; the features assumed a hideous stare; and, as exhaustion and seeming stupor followed, the intestinal contents were unconsciously discharged. In this state of things it was my first anxiety to ascertain the precise condition of the neck and mouth of the uterus. On examination, I found it *freely dilated*, there was no tumidity of its lip, the head presented, the membranes were entire. *Moderate* depletion appeared advisable, not as a directly remedial agent, but as facilitating a further seemingly perilous and questionable step. I reflected that a condition very analogous in its appearance to permanent cerebral effusion or fatal engorgement, was rapidly approaching, and that if the removal of blood were the measure of the removal of irritation, the abstraction of the first twenty ounces would either embolden me to proceed, or preclude its further flow. Accordingly, the patient was bled. The convulsions became less energetic, but the intermediate stupor more decided. The uterine efforts entirely ceased. I waited four hours, but there was only a faint renewal of contraction, consciousness remaining entirely obliterated, and the pupil rapidly dilating. The breathing, too, became loud, slow, and noisy—a complete stertorous snore. I began to think seriously of unloading the uterus; the bowels had been well cleared, not naturally alone, but by a large terebinthinate injection; croton oil had been freely administered, and there had been time to denude the scalp of its hair, and to refrigerate the head with weak spirit wash. What was to be done? I could not warrant any inference from my first experiment, that a second bleeding was likely to be more beneficial than the first; certainly there was stupor in exchange for convulsion; how far the abstraction of blood had hastened that undesirable consummation is uncertain; at least if not absolutely prejudicial, it is but negative praise to say that the symptoms were not relieved by so powerful a remedial agent. Was it probable this apparently apoplectic condition would quietly pass away, and be succeeded by calm and natural effort? I could not reason myself into this belief, and so proceeded to artificial delivery.

I experienced not the slightest difficulty in slowly and cautiously effecting the full dilatation of the os uteri; I applied Dr. Haighton's long forceps to either side of the fœtal head, and separated from the mother a living child, which (the circulation through the funis being exceedingly weak) soon after died. *But the mother recovered perfectly.* Unconscious during the operation, she expelled the placenta at the usual period; no convulsion occurred after the removal of the contents of the uterus; she gradually regained sensorial power, and the further history of her case presented no uncommon peculiarities.

Most important are the pathological principles involved in the discussion of the general question. Enough, however, is constantly occurring to stimulate investigation, and despicable even in his own estimation must that man be, to whom confided is the life of helpless, trusting *woman*, who cannot feel the solemn obligation of renewed and deliberate inquiry.—*London Lancet.*

---

*New Clinical Researches on Sanguineous Concretions formed during life in the Heart and large Vessels.* By Professor Bouillaud.—[Of two articles recently published with the above title, the first has no claim thereto, as it is simply an abstract of the opinions of Corvisart and Laennec, and of the author's previous writings on the subject of cardiac concretions. Of the second, in which M. Bouillaud endeavours to prove that the phlegmasiæ in general, and especially pneumonia, exercise a powerful influence on the formation of the coagula in question, the following summary contains the more important particulars. One case of angina tonsillaris, and fourteen of pneumonia, are briefly reported as furnishing the grounds for the doctrine.]

I cannot believe that the large masses of coagula found in the cases just related, were wholly formed some days before death, although they possessed the characters assigned to concretions of that date: they were no doubt in part formed in *articulo mortis*, and after death. The mechanism by which concretions of this latter species are produced may be compared with that regulating the coagulation of the blood removed from the vessels during life: in both cases there was a sort of plastic white fibrinous buff, so that these concretions, like the clot, may be termed *buffy*. Be this as it will, it is indubitable that in every case of acute fatal pneumonia, which has occurred in my wards within the last three years, I have found sanguineous concretions, *evidently formed in part before death*, in the heart and blood-vessels; whence I have no hesitation in establishing it as a LAW of pathological anatomy, *that these coagula exist invariably in subjects who die of well-marked acute pneumonia advanced to the second or third degree.* From our knowledge of the notable tendency of the blood to coagulate in direct idiopathic inflammations of the heart and vessels, we might have *a priori* announced that the same phenomenon would occur in the case of the prolonged febrile reaction accompanying inflammation of important organs. For, in truth, what is this febrile reaction, unless a *sympathetic* irritation of the sanguineous system? [Fever is, in the language of M. Bouillaud, merely an *angeio-carditis!*] This is so true, that in some instances we find actual inflammation of the internal membrane of the heart and aorta, instead of a simple sympathetic irritation. In pneumonia, too, the proximity of the inflamed parts to the heart and great vessels must not be forgot, as a condition facilitating the development of inflammation in the latter. [After enumerating the principal signs (none of these are novel,) by which he was enabled to diagnosticate with more or less confidence the existence of cardiac concretions in these cases, M. B. continues:] The diagnosis of sanguineous concretions of the heart and great vessels is not a matter of mere curiosity. On the contrary, it is of real importance, in establishing the prognosis of pneumonia, to ascertain whether or not the complication in question exists. This complication seriously increases the patient's danger, when the concretions are abundant, and form a considerable obstacle to the free circulation of the blood through the cavities of the heart and great vessels. [But a moment since the writer laid it down as a LAW, that these concretions invariably exist in the second and third stages of pneumonia; he now bases his favourable prognosis in inflammation of the lungs on their *absence*.] In a therapeutical point of view, also, this is an important consideration. It is not very unusual to find the pulse embarrassed, weak, and small, even when the patient himself is strong, the inflammation intense and extensive, and the heart's pulsations energetic. Under these circumstances, the majority of practitioners recommend us not to bleed; they consider the smallness of the pulse as an infallible sign of radical debility. But the truth is, that the phenomenon in question depends on the presence of coagula is the heart; bleeding is especially indicated for its removal, and, as I have ascertained in numerous instances, restores the pulse to its accustomed fulness, and raises, instead of depressing the patient's strength.—*L'Experience*, Nos. xcvi. and c., *Mai*, 1839.

---

*Remarkable Case of Ischuria Renalis, of Nine Years' Standing, with Vicarious Vomiting of Urine.* By F. L. Kreysig, M. D., Dresden.—S. B., æt. twenty-five, six years after the commencement of menstruation, began to complain of abdominal pain, general spasm, and difficulty in voiding urine and fæces, succeeded by orthopnœa, and symptoms of thoracic and abdominal inflammation, which were treated antiphlogistically. The bladder required the frequent use of the catheter. The patient suffered pain in the right knee, and was obliged to keep it drawn upwards towards the abdomen. Two years after, the legs began to swell, respiration became still more difficult, the urinary secretion *ceased*, and the patient vomited a fluid containing, according to chemical analysis, the principles of urine.

Mr. Kreysig now detected, in the right iliac region and near the spine, a tumour extending towards the liver, exquisitely painful to the slightest touch, and which the patient had noticed for four years. The bowels were invariably costive; and twice a-day (five, A. M. and six, P. M.) urinous vomitings occurred, preceded by dyspnœa, so urgent as twice to require v. s. No urine was found in the bladder. Tepid baths, and mercurial and stimulating liniments to the region of the kidney, were recommended. During the time the patient was in the baths, the skin exhaled a very fœtid odour. The tumour gradually enlarged, and grew softer.

The patient now returned home, and four months after (January 24, 1835) informed Mr. Kreysig that the tumour had daily increased, become harder and more painful, and pointed in the epigastric region. Fluctuation was now evident, and in three weeks the abscess broke, during a violent spasm, discharging a large quantity of pus, which was soon after succeeded by an increase of suffering. In about six weeks, the urinous vomiting ceased, and urine was voided by the bladder. Shortly afterwards, the pain being then more than usually intense, with an urgent desire to evacuate the bowels, the patient expelled, per anum, a mass about as large as a goose's egg, resembling fat mixed with pus, and intolerably fœtid. From this period, all abdominal swelling and pain ceased, and menstruation returned. In about three-quarters of a year, the health was entirely restored, not one of the former symptoms remaining.—*Brit. and For. Med. Review, from Hufeland's Journal*, July, 1839.

---

*Memoir on the Ophthalmia reigning in Belgium.* By M. Caffe.—This disease appeared in Belgium in 1814, but it is chiefly since 1830 that it has raged with such intensity as to have attacked an eighth of the soldiers, and in some regiments even the half. It has affected more than 100,000 individuals, and has deprived a considerable number of their vision, and left them chargeable to the state. A commission of Belgian practitioners, with the assistance of ophthalmic surgeons from Berlin and Vienna, failed in their endeavour to check the progress of this scourge, and in 1838 there were still 5,000 ophthalmic patients in an army of about 50,000 men.

In its symptoms this ophthalmia does not appear to differ materially from those with which English readers are familiar in the works of Veitch and other writers on the Egyptian epidemic; the more important part of M. Caffe's memoir is contained in his investigations into its origin and mode of extension. Reviewing the various causes to which its raging in the army has been ascribed, the author shows that it cannot be attributed to the introduction between the eyelids of the pipe-clay used in cleaning the belts and other white leather accoutrements of the soldiers, or of the powder (*tripoli*) with which their buttons and copper ornaments are polished, because the ophthalmia has affected regiments which never use those substances, and has not shown itself among other European troops who employ them in large quantity. He proves, also, that it is not due to the diet of the soldier, the abuse of spirituous drinks, the too frequent cutting of the hair, fatigue, sudden suppression of perspiration, melancholy, chlorine-fumigations used in the treatment of itch or syphilis, nor to the unhealthiness of the lodgings, to which Jungken had too hastily assigned it. He refutes especially the idea of the *compressionists*, who, with M. Vleminckx, the inspector-general, hold that the ophthalmia is produced by the pressure of the veins of the neck by a hard stock and the collar of the coat, and of those of the forehead by an inelastic heavy cap. The inefficiency of these causes M. C. shows by the fact that the French uniform before 1830, though in these respects similar to the Belgian, never produced an epidemic ophthalmia, that even in Belgium certain regiments are unaffected though dressed like the others, that the recruits who have not yet suffered from compression are often attacked on their first joining a corps in which the disease is raging, and that a complete change in the soldiers' dress has not been followed by any decrease in the disease.

M. Caffe believes that the extension of the disease depends on its contagious nature, by which it is capable of communication either by direct inoculation, by the application of the matter secreted by the diseased eye on the fingers, the linen or fluids impregnated with it, to the previously healthy eyes, or by mediate contagion or infection of the air by miasmata arising from the evaporation of the secreted fluids. Its transmission by the immediate contact of the secretion of the inflamed eye is generally admitted. The inoculation of the ophthalmia has not only succeeded on dogs, cats, and guinea-pigs, but the same result has been obtained in man, by placing the virus on an opaque cornea, or when some one with too firm a faith in non-contagion has submitted healthy eyes to the same experiment. Instances of accidental inoculation are abundant in the records of the Belgian physicians, and some of the most remarkable are cited. M. Fallot states, that on the 25th of January, 1834, there was not a single case of ophthalmia in the garrison of Namur; on that day two came into the hospital, and from that time the disease spread; two nurses were attacked, and each lost an eye. A soldier went home with an ophthalmia from the hospital at Brussels. His father, previously well, was affected a few days after with a violent conjunctivitis, and his mother and five brothers and sisters, all in succession, contracted the same disease. Another soldier went home with ophthalmia from the same hospital, and immediately after his arrival his sister was attacked. In a third similar case, the soldier's mother, brother, and sister were affected, but his father and elder sister escaped. In a fourth, the father, mother, and five brothers and sisters were all attacked. These and many other equally well-marked examples, seem to leave no doubt of the extension of the disease by direct contagion.

The miasmatic or indirect contagion is less evident; but if, says M. C., one considers the influence which an atmosphere charged with exhalations from the purulent matter must have on the conjunctiva, as well as the rapid propagation and speedy aggravation of the disease in ill-ventilated places, whatever precautions are taken against direct contagion, one will be disposed to attribute at least some share in the production of the epidemic to miasmatic infection.

The means which M. C. recommends to put a stop to the progress of the disease are such as naturally arise from the opinion he has formed, and, as it would seem, justly, that this ophthalmia, though probably originating in, and often aggravated by peculiar local circumstances, spreads from one individual to another by direct or indirect contagion. They are chiefly directed to the careful exclusion of all in whom the disease or a suspicion of it exists, from association with their comrades.—*Ibid., from Bullet. de l'Acad. Roy. de Medecine*, Jan. 15, 1840.

---

*Cases in Autoplastic Surgery—Formation of New Eyelids.* By Dr. E. Laborie.—This paper contains five successful cases of reparation of partially or completely removed eyelids, which have been observed by the author under the care of M. Jobert and M. Blandin.

In the first, of which the subject was a man æt. fifty-one, with a malignant tumour of the lower eyelid, M. Jobert removed it all from the external canthus to within two or three lines of the punctum lacrymale, exposing and removing some of the fibres of the orbicularis muscle. He then cut from the upper part of the cheek a flap of skin of an elongated triangular form, with its base slightly rounded and directed externally, and of which the truncated summit which was to serve as a pedicle was situated a little below the outer angle of the eye. This flap, which, when it was dissected up, was an inch and a half long, and half an inch wide at its outer part, was now twisted upon its pedicle and applied in the surface of the wound made by the removal of the part of the eyelid. It exactly fitted, and was fixed by three sutures. The wound on the cheek was also united by suture, and a simple slightly compressing dressing was put over the whole.

During the first few days after the operation, the wounds and the flap of skin swelled very much, but after this had ceased the adhesion was found complete. Notwithstanding two severe attacks of erysipelas, the respiration was in the end more perfect than could have been imagined; the punctum lacrymale having been preserved, there was no running of tears over the cheek, and the fibres of the orbicularis palpebrarum that remained gave the new eyelid a certain degree of natural motion.

The subject of the second case was a scrofulous woman of twenty-three, who was also under the care of M. Jobert, with ectropion of the left upper eyelid, which, from cauterization and other severe treatment, was in a completely incurable state. An incision an inch and a half long was made horizontally along the affected eyelid. A flap of skin of an oval form, and of the same size as the previous incision, was then cut out from the cheek. Its pedicle was at the level of the upper and outer part of the malar bone, and it was inclosed between two vetrical lateral incisions and an inferior incision which was slightly rounded. This portion being dissected up, was twisted on its pedicle, applied on its under surface between the edges of the incision in the eyelid, and retained there by three sutures and a simple dressing.

The operation succeeded completely; the eye, which was before constantly exposed, was fairly covered by the new eyelid, which, by the actions of what remained of the orbicularis muscle, performed all the natural motions. A second operation of a somewhat similar kind, was subsequently performed with the same success for the reparation of a loss of substance in the situation of the eyebrow. In this case, a portion of the skin from the temple, which was in part covered with hair, was taken for the flap; the hairs continued to grow, and by cutting them to a proper length they formed an excellent substitute for the lost eyebrow.

The third case was one of ectropion of the lower eyelid from a burn, which was treated in the same manner as the second, with the same favourable result.

The fourth was that of a young woman who, from acute inflammation and sloughing, had lost the whole of the left upper eyelid except its free border, which contained the eyelashes, the cartilage, and the meibomian glands, from which a sticky fluid constantly running had fixed this remnant of the upper eyelid to the edge of the lower. There was acute inflammation of the exposed surface of the eye.

M. Blandin cut a flap of skin from the forehead, of an oval form, with its pedicle at the root of the nose. Then having cut off the edges of the ulcerated aperture, he turned down the flap over it, and fastened it to them by sutures. Notwithstanding a very severe attack of erysipelas, the flap adhered perfectly at all parts. The new eyelid had lashes, and even a slight power of motion, though the orbicularis had been entirely destroyed; for a portion of the frontalis muscle turned down with the flap of skin, preserved its contractility, and by the transverse position in which its fibres were now placed, it enabled the eyelid to be slightly raised.

The last case was one of ectropion of the lower eyelid, which was treated like the second and third, by fitting a flap of skin into the space between the edges of an incision made along the everted part. It was successful, and the eye obtained almost complete protection from the new eyelid.—*Ibid., from Gazette Medicale*, Janvier 13, 1840.

---

*Cases of Poisoning by Arsenic, Sulphuric Acid, and Muriate of Mercury.* By ALEXANDER WATSON, F. R. S. C. E., Surgeon to the Royal Infirmary, &c. &c.—The three following cases of poisoning from arsenic, sulphuric acid, and muriate of mercury, illustrate the effects of those substances, and are therefore deserving of being recorded.

CASE 1. *Poisoning from Arsenic.*—Peter Baulks, a collier, about 45 years of age, residing at Dewartown, in the parish of Borthwick, near Edinburgh, seemed to have lived unhappily with his wife, who, in revenge for a blow she had received from him, purchased arsenic and administered a quantity of it to him along with salts, on the morning of the 28th April, 1835, of which he died in nine hours after. Mrs. Baulks having been tried on the 13th July, 1835, and found guilty, was afterwards executed for the murder thus committed.

Baulks took the dose of salts and other ingredients administered to him by his wife, about ten o'clock on the morning of the 28th April. He was very soon afterwards seized with vomiting, pain of the bowels, and purging, accompanied with great prostration of strength. This illness, Mrs. B. said, was the cholera, but would not hear of a doctor being called. To the neighbors who had come into see him, Baulks said it was the dead scour that was upon him, and blamed his wife for the medicine she had given him. He continued ill, and gradually became weaker till seven o'clock P. M., when he died, having been ill nine hours.

At the request of the sheriff of the county, I proceeded with my assistant, Mr. Rae, now surgeon at Stirling, to inspect the body of Baulks, on the third of May, in order to report as to the cause of his death.

On opening the chest, we found the lungs to be affected with the blackness or spurious melanosis so frequently observed in the lungs of colliers.

When the viscera of the abdomen were exposed, no unusual appearance was observed externally. There was no appearance of inflammation at any part either of the stomach or bowels. The whole of the intestinal canal, from the tongue downwards, was carefully removed from the body without being opened, and the escape of the contents being at the same time prevented by ligatures.

Upon the following day, these parts were taken by me to the college, in order that they might be examined, and their contents analysed by Professor Traill, as well as Mr. Rae and myself.

The glands of the mucous membrane at the root of the tongue, and at each side of the larynx, were much enlarged, and the whole of the membrane presented an appearance of increased vascularity and unusual irritation.

On cutting open the œsophagus and stomach, their internal surface was thickly coated with mucus. The stomach contained about a pound of viscid bloody fluid, which was carefully removed into a clean vessel. A few small white particles were seen among the mucus, with which the inner surface of the stomach was coated, and were carefully removed by means of a clean piece of silver, and placed upon a glass plate. The mucous membrane lining the stomach, was generally of a deep red colour, with the exception of two patches, which exhibited a greenish or ash-gray colour. Near to the cardiac orifice, a portion of the membrane was abraded. About three feet of the small intestines were also laid open, but the mucous membrane presented nothing unusual in appearance, and no white particles could be detected upon it.

By boiling a portion of the fluid contents of the stomach, and adding some distilled acetic acid, while hot, the animal matter was coagulated. When this was separated by filtration, the fluid was tested by nitrate of silver, the ammoniacal nitrate of copper, and sulphuretted hydrogen; but the presence of arsenic could not be distinctly traced in it.

The small white particles which had been picked from the inner surface of the stomach, being collected and put into a tube, and boiled in distilled water with the addition of a little potash, were almost entirely dissolved. The animal matter being then separated from the liquor by acetic acid, and the filtered liquor subjected to a stream of sulphuretted hydrogen gas, the yellow colour given to the fluid, and the orange-coloured flaky precipitate produced by the presence of arsenic, soon became manifest. The excess of sulphuretted hydrogen having been removed by boiling, the liquid was evaporated to dryness in a small glass cup. When the precipitate became dry, it was carefully scraped from the glass cup, and introduced into a small glass tube along with some black flux. Upon the application of heat, metallic arsenic was obtained.

Upon boiling a portion of the more viscid and solid contents of the stomach, which remained at the bottom of the vessel into which they had been put, in distilled water and potash, treating this mixture with acetic acid in excess, and subjecting it to a stream of sulphuretted hydrogen, a copious arsenical precipitate was obtained, which was further demonstrated by the tests of reduction and oxidation.

In this case, therefore, we had no difficulty in certifying that death had been occasioned by arsenic.

CASE 2. *Suicide from Sulphuric Acid and Incision of Throat.*—Widow H., aged 60, a stout corpulent woman, was brought into the Royal Infirmary on the evening of the 7th February, 1838, having her throat extensively cut, and died shortly after admission. It was afterwards ascertained that she had purchased two ounces of sulphuric acid, and swallowed it about a a quarter of an hour before cutting her throat. After having taken the acid, she was seen writhing about in great pain; she had then put one of her late husband's razors into her pocket, and left the house to cut her throat. She inflicted the incisions on arriving at the street, was immediately seen and conveyed to the Infirmary, which was close by. This individual, therefore, probably did not live above half an hour after taking the acid into the stomach.

The inspection of the body took place at one o'clock on the 9th February; and as no circumstance had then come to my knowledge leading to the supposition of poison having been swallowed, my attention was at first directed chiefly to the examination of the wound and the state of the heart and the blood-vessels.

The external wound across the throat was immediately under the lower jaw, and about five inches in length. It divided, by a pretty clean incision, the integuments, upon which there were marks of four incisions,—the larynx between the *os hyoides* and thyroid cartilage, the anterior part of the *platysma myoides*, the anterior border of the sterno-mastoid, the omohyoid, the sterno-hyoid, and sterno-thyroid muscles, were divided. The epiglottis was cut across transversely at its base, as also the anterior half of the muscular parietes of the pharynx. The anterior facial vein, at its junction with the common facial, the pharyngeal and lingual veins of both sides, at their termination in the internal jugular, and the internal jugular vein of the left side, were freely divided. The superior subcutaneous cervical plexus of nerves, and the superior laryngeal nerves, were also cut across. The carotid arteries were entire. The trachea and larger bronchial tubes were filled with coagulated blood. The quantity of congested blood in the lungs was considerable. With the assistance of Dr. Handyside, I secured by ligature all the veins and arteries connected with the heart. There was a large globule of gaseous fluid, and some blood in the *vena anonyma*, and a quantity of the same could be pressed from the right ventricle of the heart into the pulmonary artery, so as to distend it. The heart was flaccid and almost empty. Upon removing the heart from the body, and opening it carfully under water, Dr. Handyside collected in a receiver about two drachms, by measure, of gaseous fluid, which Dr. Fyffe was requested to examine, and obligingly favoured us with the following analysis of it. "The gas you left with me yesterday is mainly atmospheric air, containing the usual proportions of oxygen and nitrogen, with a slight admixture of carbonic acid. The acid gas seemed to be in greater proportion than usually exists in air, but, owing to the minute quantity of gas left with me, I could not ascertain its proportion with precision."

On opening the cavity of the abdomen, the peritoneum lining the anterior part of its parietes was extensively corroded, charred, and decomposed, by which the fibres of the muscles beneath it were laid bare. The omentum was extensively destroyed in a similar manner. The whole of the peritoneal covering of the intestines, which was in contact with the anterior parietes of the abdomen, was changed to a bluish gray colour. The small intestines were exceedingly contracted. About three-fourths of the stomach were wanting, having been completely dissolved and decomposed; and the anterior part of the small arch which remained was softened, as if it had been partially decomposed by long maceration in water. The mucous membrane had a blackened and charred appearance. The mucous membrane lining the œsophagus was black. The upper part of the duodenum was also softened and corroded. The contents of the stomach, mixed with oily fluid, were extravasated into the abdominal cavity, as also the remains of the decomposed parts. The erosion of the peritoneum was chiefly confined to the right side, at and below the stomach. The peritoneal covering of the liver was condensed and firm to the touch, of a gray colour, having a corroded appearance, and some black patches upon it.

The coats of that part of the great arch of the colon in contact with the stomach, were also corroded.

The blood-vessels of the parts affected were distended with hardened dark-coloured blood.

After macerating the remaining part of the stomach in water, and this being afterwards filtered, a white precipitate was obtained from a solution of muriate of baryta added to it, thereby giving evidence of the presence of sulphuric acid.

As already stated, I had no knowledge at the time of the dissection that poison of any kind had been swallowed, but when examining the abdomen, and seeing the above state of the parts, I observed to those beside me that the effects which the fluid in the abdomen had upon the fingers, of smarting cnd corrugation of the skin, which I experienced, was very much the same as those of sulphuric acid somewhat diluted.

Whether the immediate cause of death in this case was owing to hæmorrhage from the wound in the throat; suffocation from blood in the air passages; the admission of air into the heart by the veins; or to the sulphuric acid swallowed, it is not easy to determine. Probably more than one of these contributed to produce the fatal result. It is also difficult to determine how much of the disorganization produced by the sulphuric acid, took place during life, and what changes took place after death. Upon these points I shall not at present venture to speculate.

CASE 3. *Of Poisoning from Corrosive Muriate of Mercury.*—The following case came under my notice, being the subject of judicial investigation.

Alexander Bell, aged 47, a dyer from Musselburgh, was admitted into the Royal Infirmary, under the care of Dr. Shortt, on the 3d September, 1838. The following is the statement of his case contained in the Journal of the Hospital.*

"States, that on the 26th ultimo, feeling rather unwell, he applied to an apothecary for a calomel powder, from whose wife he received about a teaspoonful of a heavy white powder, which he took in tea. While swallowing it, felt an acute burning sensation in the throat and stomach; and immediately after, was seized with stiffness of the jaws, purging and vomiting, great pain in the bowels, accompanied with cramps, stools dark-coloured and bloody, pain in the mouth continued with ptyalism, and occasionally the pain in the bowels was very severe."

"On admission, complains of great weakness; the jaws stiff and swollen; there is a good deal of enlargement of the parotid gland on the right side; the gums swollen and spongy; there is great ptyalism, with strong mercurial fetor of the breath; pulse 64, and very weak.

"Sept. 5th. Feels the mouth considerably easier.

"Sept. 6th. During the night, and while asleep, discharged from the mouth without vomiting, a considerable quantity of fluid blood, and feels weaker this morning.

"Sept. 7th. Discharge of blood returned last night, and the patient feels much weaker this morning. Bowels rather open, but no blood in the stools.

"*Vespere.* The bleeding has returned, and strength failing.

"Sept. 8th. Has felt some benefit from the lotion, but the hæmorrhage has not been completely arrested by it. Sleeps a good deal; pulse 100, scarcely to be felt; whole appearance denoting extreme exhaustion.

"Sept. 9th. The patient passed about six pounds of blood by stool, about six o'clock last night, and died a few minutes afterwards."

In consequence of my being requested by the legal authorities to examine the body of Bell, and report as to the cause of his death, I did so along with Dr. Shortt, on the 9th of September, 1838, and the following are the morbid appearances which we observed.

Ulceration of the gums and mucous membrane of the fauces and œsophagus in patches of various sizes. The papillæ at the root of the tongue were much enlarged. There was no redness or increased vascularity of the peritoneum at any part. The stomach and bowels seemed to contain only grumous blood and mucus. When this was removed by washing, the mucous membrane of the stomach was much more red than usual, from increased vascularity. At the great arch of the stomach this was increased to a very dark-red colour, probably by being dyed by the presence of blood in it. About two inches from the cardiac orifice of the stomach, and extending downwards to its greater arch, there was an extensive ul-

* An account of this case was published in this Journal for January, 1839, by a Mr. Alexander Wood, surgeon, who happened at the time to be acting as clerk to Dr. Shortt in the Royal Infirmary. Besides the impropriety of publishing the case while it was undergoing judicial investigation, contrary to the regulations of the Managers of the Infirmary, and without my permission, allusion is here made to the contents of an official report drawn up by Dr. Shortt and myself, which Mr. Wood was allowed in confidence to read over.

ceration upon the inner surface, of a triangular shape, each side of which measured about three inches. Several sloughs formed of the mucous membrane were hanging from the muscular coat, which was exposed in the process of separation at this part. At several other places there were ulcerations forming by the separation of sloughs from the mucous membrane, but of much smaller size. The margins of these ulcerations had a yellowish-green appearance, and lymph appeared to be effused upon the ulcerated surface. Appearances somewhat similar, but to a much less extent, were observed on the mucous membrane of the colon. The glands or mucous papillæ of the rectum were much enlarged.

The report, to which allusion has been made, which was drawn up by Dr. Shortt and myself, and transmitted to the legal authorities, stated shortly the morbid appearances found on dissection. It also contained an account of the symptoms under which Bell had laboured since the 26th of August; and stated as our opinion, that, considering these symptoms in connection with the morbid appearances, there appeared to us "no doubt that the illness and death of the said Alexander Bell had been caused by his having swallowed a large quantity of some of the more corrosive preparations of mercury."

We have preserved some of the contents of the stomach and bowels, as also some of the blood, for chemical analysis; but, as was anticipated, without any satisfactory result, partly from the length of time the patient lived after the poison was taken, and partly from the difficulty and uncertainty which attends the detection of mercury in the body.

It was afterwards ascertained that Bell had himself written a note for corrosive sublimate of mercury, which he sent to the shop from which it had been obtained, as a remedy for some complaint.

Having stated fully and minutely the facts connected with these cases of poisoning, I purposely refrain from entering into any speculative remarks or general inferences, because I consider general conclusions drawn from individual cases to be of no value. The cases are therefore detailed in order that they may be compared with other cases of the same kind, generalized, and commented upon.

There is, however, one circumstance which occurred in the first and third of the cases above described, which is worthy of notice in a legal point of view. The circumstance to which I allude is, that the arsenic and corrosive sublimate were very loosely dispensed and sold to the individuals who asked for them, by unqualified persons, (females,) in the absence of the masters of the establishments. Had greater caution been employed in the sale of these articles, the very disastrous consequences to which they led might possibly have been prevented. These, and other similar cases, which are of far too frequent occurrence, make it very necessary that some restriction should, *by a legal enactment*, be put upon the sale of such deadly drugs.

*Edinburgh Med. and Surg. Journal.*

---

*Mortality in the Prussian Army for the year* 1838.—During the year 1838, 142,234 patients have passed through the Prussian army hospitals, of which number, 4,992 were remaining over from the month of December, 1837. The number of patients admitted each month of the year, arranged according to the relative proportion of admissions, gives the following result: In the month of January, 14,086 patients were admitted; May, 13,742; June, 12,683; March, 12,281; February, 12,278; August, 11,263; July, 11,100; April, 10,811; December, 10,273; September, 10,216; November, 9,989; October, 8,450; the total amount of admissions for the year 1838 being 137,242.

Of the total number, 142,234, there were 135,925 dismissed cured; and 1,333 died; 429 were incurable, and no mention is made of 8. At the end of the year 1838, there remained in the hospitals 4,539 patients. In the tables detailing the causes of death, it is shown that 456 died from nervous fevers; 351 from phthisis; 171 from inflammatory fevers; 82 from dropsy; 32 from delirium tremens; 41 from old age; 19 from apoplexy, &c. These tables also show that there were the astonishing number of 67 deaths from suicide, of which 48 were by fire arms, 13 by hanging, 4 by drowning, and 2 by cutting the throat.—*L'Experience*, 25th December, 1839.

---

*New Treatment of Cancer.*—M. Jobert has endeavoured to check the progress of this malady, by tying all the vessels and dividing all the nerves which are distributed to the affected part. His efforts, however, have not been crowned with success.—*L'Experience*, l. c.

---

*Case of Uterine Hæmorrhage in which the blood escaped through the Fallopian Tubes.* By W. F. Barlow, Esq.—A young woman, 22 years of age, who had miscarried at the sixth month, with much flooding, had an attack of *purpura hæmorrhagica*, from which she died five days after abortion had taken place. On dissection, a quantity of blood was found to have been infused into the abdomen and pelvis, some of which was coagulated; and as clots were found projecting from the fimbrated extremities of the Fallopian tubes, from which it was evident they had been expelled, the author infers that uterine hæmorrhage had taken place through these tubes into the abdomen. The uterus was less than the size it generally assumes a week after delivery, and a coagulum of blood partly occupied the neck of the organ.—*London Medical Gazette*, November 11, 1839.

